# Clinical and molecular overlap between nucleotide excision repair (NER) disorders and *DYRK1A* haploinsufficiency syndrome

**DOI:** 10.3389/fnins.2025.1554093

**Published:** 2025-03-26

**Authors:** Nicolas Le May, Jérémie Courraud, Imène Boujelbène, Cathy Obringer, Tomoo Ogi, Alan R. Lehmann, Fanny Laffargue, Daphné Lehalle, Seiji Mizuno, Shehla Mohammed, Clothilde Ormières, Marjolaine Willems, Vincent Laugel, Nadège Calmels

**Affiliations:** ^1^Laboratoire de génétique médicale, Faculté de médecine de Strasbourg, Strasbourg, France; ^2^Equipe Génétique et physiopathologie de maladies neurodéveloppementales et épileptogènes, IGBMC, Illkirch, France; ^3^Laboratoires de diagnostic génétique, Hôpitaux Universitaires de Strasbourg, Strasbourg, France; ^4^Research Institute of Environmental Medicine, Nagoya University, Nagoya, Japan; ^5^Genome Damage and Stability Centre, University of Sussex, Brighton, United Kingdom; ^6^Service de génétique médicale, CHU de Clermont-Ferrand, Clermont-Ferrand, France; ^7^Service de Génétique, Groupe Hospitalier Pitié Salpêtrière, Paris, France; ^8^Central Hospital, Aichi Developmental Disability Center, Kamiya, Kasugai, Japan; ^9^South East Thames Regional Genetics Service, Guy's Hospital, London, United Kingdom; ^10^Service de Génétique Clinique, Hôpital Necker-Enfants Malades, Paris, France; ^11^Equipe Maladies Génétiques de l'Enfant et de l'Adulte, CHU de Montpellier, Montpellier, France; ^12^Service de pédiatrie, Hôpitaux Universitaires de Strasbourg, Strasbourg, France

**Keywords:** *DYRK1A* gene, nucleotide excision repair (NER), Cockayne syndrome, trichothiodystrophy, *ERCC6/CSB* gene, *ERCC8/CSA* gene

## Abstract

Nucleotide excision repair (NER) disorders are genetic conditions caused by defects in the pathway responsible for repairing DNA lesions due to UV radiation. These defects lead to a variety of heterogeneous disorders, including Cockayne syndrome (CS) and trichothiodystrophy (TTD). In this study, we report 11 patients initially suspected of having CS or TTD who were ultimately diagnosed with *DYRK1A* haploinsufficiency syndrome using high-throughput sequencing. Comparing clinical presentations, we observed that *DYRK1A* symptoms overlapped with CS, with shared features such as intellectual disability and microcephaly, systematically present in both disorders and other common symptoms including feeding difficulties, abnormal brain imaging, ataxic gait, hypertonia, and deep-set eyes. However, distinctive features of *DYRK1A* syndrome, such as severely impaired language, febrile seizures, and autistic behavior or anxiety, helped differentiate it from CS, which typically manifests with severe growth delay, bilateral cataracts, and pigmentary retinopathy. Among the cohort, three patients carried novel *DYRK1A* variants, including two truncating and one in-frame variant p.Val237_Leu241delinsGlu whose pathogenicity have been confirmed through functional analysis of DYRK1A protein. While previous research has implicated DYRK1A in DNA repair, with *DYRK1A* being one of the most downregulated genes in CS cells, our study found that DYRK1A patient-derived cell lines did not exhibit NER defects and did not share the CS transcriptomic signature. These findings suggest that if clinical symptoms overlap stems from common molecular disruptions, *DYRK1A* is involved downstream of the CS genes. This research highlights the importance of considering *DYRK1A* haploinsufficiency syndrome in the differential diagnoses for NER disorders.

## 1 Introduction

Nucleotide excision repair (NER) is a critical DNA repair mechanism that protects cells from a variety of DNA damages, such as those caused by ultraviolet (UV) radiation and chemical mutagens (Nieto Moreno et al., 2023). Beyond their role in DNA repair, NER proteins also influence the dynamic regulation of transcription and chromatin structure, which are essential for maintaining genomic stability and proper gene expression. Defects in the NER pathway lead to a variety of distinct, heterogeneous disorders, such as xeroderma pigmentosum (XP), Cockayne Syndrome (CS), and Trichothiodystrophy (TTD), all with autosomal recessive inheritance. Whereas XP is a genodermatosis mainly characterized by a predisposition to sunlight-induced skin pigmentation changes and skin cancers, as well as neurological problems in 30–50% of cases, CS and TTD are neurodevelopmental and subsequently neurodegenerative disorders leading to progressive microcephaly, growth retardation, developmental delay, neurological and neurosensory impairments and skin and/or hair abnormalities. CS encompasses a wide spectrum of severity, with the classic form, known as type I, onset occurring before the age of 2. The genetic abnormalities underlying these diseases are multiple and complex, with mutations in the same gene leading to different conditions (Ferri et al., 2020). Moreover, the existence of combined forms, such as XP/CS, further complicates the diagnosis. Historically, the diagnosis was confirmed by DNA repair activity testing on primary fibroblasts, such as recovery of RNA synthesis (RRS) and Unscheduled DNA synthesis (UDS) assays (Mayne and Lehmann, 1982). More recently, the diagnosis of these heterogeneous conditions has significantly benefited from the use of high-throughput sequencing techniques, as formerly demonstrated by sequencing a panel of NER genes (Calmels et al., 2016). At the end of these investigations, however, some patients with suspected DNA repair disease remain without any identified molecular cause. These patients may carry variants in unexplored regions of NER genes such as promoter or deep intronic regions (Schalk et al., 2018). They may also be clinical phenocopies (so called NER-like patients) whose molecular origin lies in other genes than those of the NER pathway.

*DYRK1A* haploinsufficiency syndrome (OMIM# 614104) is a neurodevelopmental disorder caused by mutations in the *DYRK1A* gene, which encodes a protein kinase essential for brain development. It is characterized by a range of cognitive and developmental impairments, including microcephaly, intellectual disability, motor delay, speech difficulties, feeding problems, autism spectrum traits, seizures, and dysmorphic features. Studies have reported that a majority of patients exhibit atopic skin or skin that appears thin and translucent (Courraud et al., 2021). Some individuals may also present with fine hair texture. Interestingly, *DYRK1A* has been previously reported as one of the most down-regulated ATF3-dependent genes in CS fibroblasts, suggesting a potential functional link between NER proteins and DYRK1A (Epanchintsev et al., 2020).

Here we report on 11 patients, initially suspected of NER disorders, who were finally diagnosed with *DYRK1A* haploinsufficiency syndrome through further genetic investigations. Extended genetic investigations in NER-like patients enabled us to identify the first four patients. In a second phase, we identified an extra *DYRK1A* patient by a retrospective analysis of our in-house cohort of NER-like patients. Finally, six additional NER-like patients with a *DYRK1A* variant were enrolled through an international collaboration with British and Japanese teams involved in diagnosing NER disorders. We will discuss the phenotypic overlap between NER disorders and *DYRK1A* haploinsufficiency syndrome and attempt to explore the potential underlying molecular mechanisms of these common features.

## 2 Methods

### 2.1 Patient recruitment and molecular analysis

All patients included in this study gave written consent for genetic testing. *DYRK1A* coding sequence was analyzed through Sanger sequencing, gene panel sequencing or exome sequencing. *DYRK1A* variants are named according to GRCh38 version of genome assembly, NM_001396.5 transcript and NP_001387.2 protein. New variants have been registered in ClinVar Database.

### 2.2 DNA repair functional assays

Primary fibroblasts from four individuals with *DYRK1A* variant (patients 1, 2, 4 and 5) were available for DNA repair functional assays and were compared to fibroblasts from a healthy control, from a Cockayne individual (carrying pathogenic variants in the *ERCC8/CSA* gene) for RRS assay and from a xeroderma pigmentosum individual (carrying pathogenic variants in the *ERCC4/XPF* gene) for UDS assay. Cells were stored in the registered biobank DC-2014-222 CPP 09/40. Recovery of RNA synthesis (RRS) and unscheduled DNA synthesis (UDS) following UVC irradiation of the primary fibroblasts were performed using fluorescent non-radioactive assays as previously described (Jia et al., 2015; Calmels et al., 2016).

### 2.3 ATF3 and ATF3-dependent genes expression levels

Expression profiles were studied on primary fibroblast cultures from two controls, three CSB patients with *ERCC6* mutations, three CSA patients with *ERCC8* mutations and from patients 1, 2, 4 and 5. Fibroblasts were seeded on 10 cm plates using 1×10^6^ cells. Twenty-four hours later, cells were UV-irradiated (20 J/m^2^) and then incubated for 24 h in DMEM (1 g/L glucose) with GLUTAMAX (Life Technologies, Inc., Rockville, MD) supplemented with 10% of Fetal Calf Serum (FCS) and Penicillin (100 UI/mL) + Streptomycin (100 μg/mL). Total RNA was extracted from the fibroblasts using the GenElute Mammalian Total RNA Miniprep kit (Sigma-Aldrich) and reverse transcribed with SuperScript IV reverse transcriptase (Invitrogen). Quantitative PCR was performed using SYBR Green Master Mix and the CFX384 Real-time system (Biorad). The primer sequences for genes analyzed by qPCR are listed in Supplementary Table S1. mRNA expression of the various genes was calculated as the ratio of values obtained from irradiated versus non-irradiated cells, normalized to the housekeeping *GAPDH* mRNA. Biological triplicates and technical duplicates were analyzed. T-test was performed to compare the expression level of each gene in CSA, CSB and *DYRK1A* patients to the expression level in control cell lines, applying Welch’s correction.

### 2.4 *In vitro* analysis of variant effect on DYRK1A protein

Level, cellular localization, and kinase activity of DYRK1A mutant proteins were studied as previously reported by our team (Courraud et al., 2021). Briefly, wild-type (WT) and mutant DYRK1A proteins were overexpressed in three different cell lines (HEK293, HeLa, COS1) using *DYRK1A* expression plasmids. Protein level was studied in the three cell types by Western blot. One-way ANOVA with multiple comparison test was performed to compare the level of mutant DYRK1A proteins to the level of WT DYRK1A protein, applying Bonferroni’s correction. Cellular localization was studied in HeLa cells by immunofluorescence. Chi-square test was performed to compare localization of mutants and wild-type DYRK1A proteins. DYRK1A autophosphorylation activity was evaluated in HEK293 cells using the ratio between Tyr321 DYRK1A and total DYRK1A protein. One-way ANOVA test was performed to compare mutants to wild-type DYRK1A proteins.

## 3 Results

### 3.1 Identification of DYRK1A pathogenic variants in individuals clinically suspected of NER disorders

We set up a cohort of 11 patients initially suspected of having an NER disorder (CS or TTD) but carrying a pathogenic variant in the *DYRK1A* gene (NM_001396.5) (Table 1).

**Table 1:** List of variants identified in *DYRK1A* in individuals initially suspected of nucleotide excision repair (NER) disorder.

	Gender	Origin	RRS	UDS	GRCh38 (Chr21)	c. (NM_001396.5)	p. (NP_001387.2)	Type	Inheritance
Patient 1	M	France	N	N	g.37493099G > A	c.1034G > A	p.(Trp345Ter)	Ns	*de novo*
Patient 2	F	France	N	N	g.37490297C > T	c.787C > T	p.(Arg263Ter)	Ns	*de novo*
Patient 3	F	France	NA	NA	g.37486561del	**c.611del**	**p.(Val204GlyfsTer7)**	Fs	*de novo*
Patient 4*	F	France	N	N	g.37496251dup	c.1232dup	p.(Arg413ThrfsTer10)	Fs	*de novo*
Patient 5	F	France	N	N	g.37490220_37490232delinsA	**c.710_722delinsA**	**p.(Val237_Leu241delinsGlu)**	Infr.	not in mother
Patient 6	F	UK	NA	NA	g.37490424_37490429del	c.914_919del	p.(Ile305_Asp307delinsAsn)	Infr.	*de novo*
Patient 7	M	UK	N	N	g.37478275del	c.302del	p.(Ile101ThrfsTer49)	Fs	NA
Patient 8	NA	USA	N	N	g.37505352C > T	c.1309C > T	p.(Arg437Ter)	Ns	NA
Patient 9	F	UK	N	N	g.37490424dup	c.914dup	p.(Val306SerfsTer2)	Fs	NA
Patient 10	F	UK	N	N	g.37480659C > T	c.349C > T	p.(Arg117Ter)	Ns	NA
Patient 11	M	Japan	NA	NA	g.37490213dup	**c.703dup**	**p.(Cys235LeufsTer5)**	Fs	*de novo*

F, female. Fs, frameshift. Infr., in-frame. M, male. N, normal. NA, not available. Ns, nonsense. RRS, recovery of RNA synthesis assay. UDS, unscheduled DNA synthesis assay. In bold: variant never described before and registered in ClinVar database: c.611del (ID 3391808), c.710_722delinsA (ID 3392473) and c.703dup (ID 3391807). *Already published patient (Bronicki et al., 2015; Courraud et al., 2021).

Patients 1 to 4 are French patients initially addressed for an NER disorder (CS or TTD). Sanger sequencing of the 2 main CS genes, *ERCC6* and *ERCC8,* or high-throughput sequencing (HTS) of a panel of 16 genes involved in the NER pathway (Calmels et al., 2016), did not identify any pathogenic variant. Extended genetic analyses using HTS of a large panel of genes involved in neurodevelopmental disorders have revealed the presence of *de novo* pathogenic variants in the *DYRK1A* gene.

In order to identify additional cases, we analyzed *a posteriori* our in-house cohort of 233 “NER-like” patients. Based on clinical records, we selected individuals with microcephaly, intellectual disability, global development delay and impaired language. Patients presenting with photosensitivity or pigmentary retinopathy, symptoms never described in *DYRK1A* patients, were excluded. Among the three selected patients, *DYRK1A* sequencing using high-throughput or Sanger sequencing, revealed an in-frame deletion/insertion variant, p.(Val237_Leu241delinsGlu) in one patient (patient 5). The variant was not maternally inherited but father’s DNA was not available for testing.

Finally, through an international collaboration with British and Japanese diagnosis teams involved in NER disorders, six additional NER-like patients carrying a loss-of-function *DYRK1A* variant identified by exome sequencing were recruited (patients 6 to 11). The variant occurred *de novo* in two of them, while parental DNA was not available for the remaining cases.

### 3.2 Clinical description

We reviewed the clinical manifestations of the patients (Table 2; Supplementary Table S2). Consistent with previous reports (Bronicki et al., 2015; Ji et al., 2015; van Bon et al., 2016; Courraud et al., 2021) recurrent features include psychomotor delay evolving to intellectual disability, prenatal and postnatal moderate growth delay (median height: −2.6 ± 1.4 DS), microcephaly and major speech impairment, feeding difficulties, seizures, autistic traits and anxiety, delayed gross motor development with unstable gait, brain magnetic resonance imaging (MRI) abnormalities including enlarged ventricles, cerebral atrophy and cerebellar hypoplasia, and distinctive facial features. We found skin and hair anomalies especially atopic dermatitis and thinning hair. When clinical data were available, we measured the clinical DYRK1A score (Courraud et al., 2021). The range and mean scores (from 14 to 18.5, mean = 16.2) is similar to those of the published cohort (from 13 to 18.5, mean = 15.5) and all evaluated patients have a score above the discriminant threshold of 13 (Supplementary Table S3).

**Table 2:** Comparative clinical characteristics between classical descriptions of Cockayne and *DYRK1A* syndromes (percentages are derived from existing literature) and this *DYRK1A* cohort (numbers reflect occurrences of each symptom among the patients for whom the information was available).

	Cockayne syndrome, literature (Nance and Berry, 1992; Wilson et al., 2016; Laugel, 1993)	*DYRK1A* syndrome, literature (Courraud et al., 2021; van Bon et al., 1993)	*DYRK1A* syndrome, present cohort
Growth			
Intrauterine growth retardation	15%	frequent	8/8
Postnatal failure to thrive (weight < -2SD)	100%	49%	6/7
Postnatal short stature (length or height < -2SD)	100%	44%	6/8
Neurology			
Microcephaly	100%	94–100%	10/10
Intellectual disability	100%	100%	8/8
Speech impairment	frequent	100%	4/4
Epilepsy	23%	65–88%	5/6
Autistic traits	NR	46–89%	3/4
Motor delay	frequent	82–88%	6/8
Ataxic gait	frequent	64–73%	4/5
Brain imaging			
White matter / myelination anomalies	38%	frequent	4/4
Brain calcifications	55%	NR	NR
Cerebral atrophy	frequent	frequent	4/4
Cerebellar atrophy	frequent	frequent	3/4
Atrophy of the corpus callosum	frequent	frequent	NR
Vision and hearing			
Optic nerve hypoplasia	frequent	rare	1/1
Cataracts	48.5%	rare	2/4
Pigmentary retinopathy	43%	NR	1/4
Hearing loss	84%	rare	2/3
Dermatology			
Clinical photosensitivity	78%	NR	NR
Skin cancer predisposition	NR	NR	NR
Atopic or translucent skin	NR	68%	3/4
Thinning hair	46%	NR	5/5
Other symptoms			
Characteristic facial features / deep-set eyes	100%	90%	10/10
Dental caries / enamel hypoplasia	46%	NR	NR
Morphological dental anomalies	frequent	17%	1/1
Scoliosis / kyphosis	48%	NR	1/3
Urogenital anomalies	NR	40%	1/2
Feeding difficulties (in infancy)	48%	88–93%	5/5
Abnormal liver function	63%	NR	NR

Gray boxes highlight to the cardinal signs included in the published diagnostic scoring systems for Cockayne (Spitz et al., 2021) and *DYRK1A* syndromes (Courraud et al., 2021). NR, not reported.

### 3.3 Molecular overlap between variants identified in individuals with NER-like disorders and *DYRK1A* haploinsufficiency syndrome

Among the 11 *DYRK1A* variants described in this cohort, nine were loss of function (five frameshift variants and four nonsense variants) distributed throughout the protein, while the remaining two were in-frame deletion/insertion variants affecting amino acids located within the kinase domain (Table 1). Interestingly, only three of these variants have not been previously described, whereas the others have already been reported in individuals with *DYRK1A* syndrome or unspecified neurodevelopmental delays. Notably, two of the variants, p.(Arg437Ter) and p.(Arg117Ter), are the most recurrent variants associated with DYRK1A syndrome, with at least eight submissions in ClinVar and likely more cases identified but not reported. The *DYRK1A* patients in our cohort, selected based on their similarity to NER patients, exhibit clinical and molecular characteristics closely resembling those of previously published *DYRK1A* cases.

**Figure 1:**
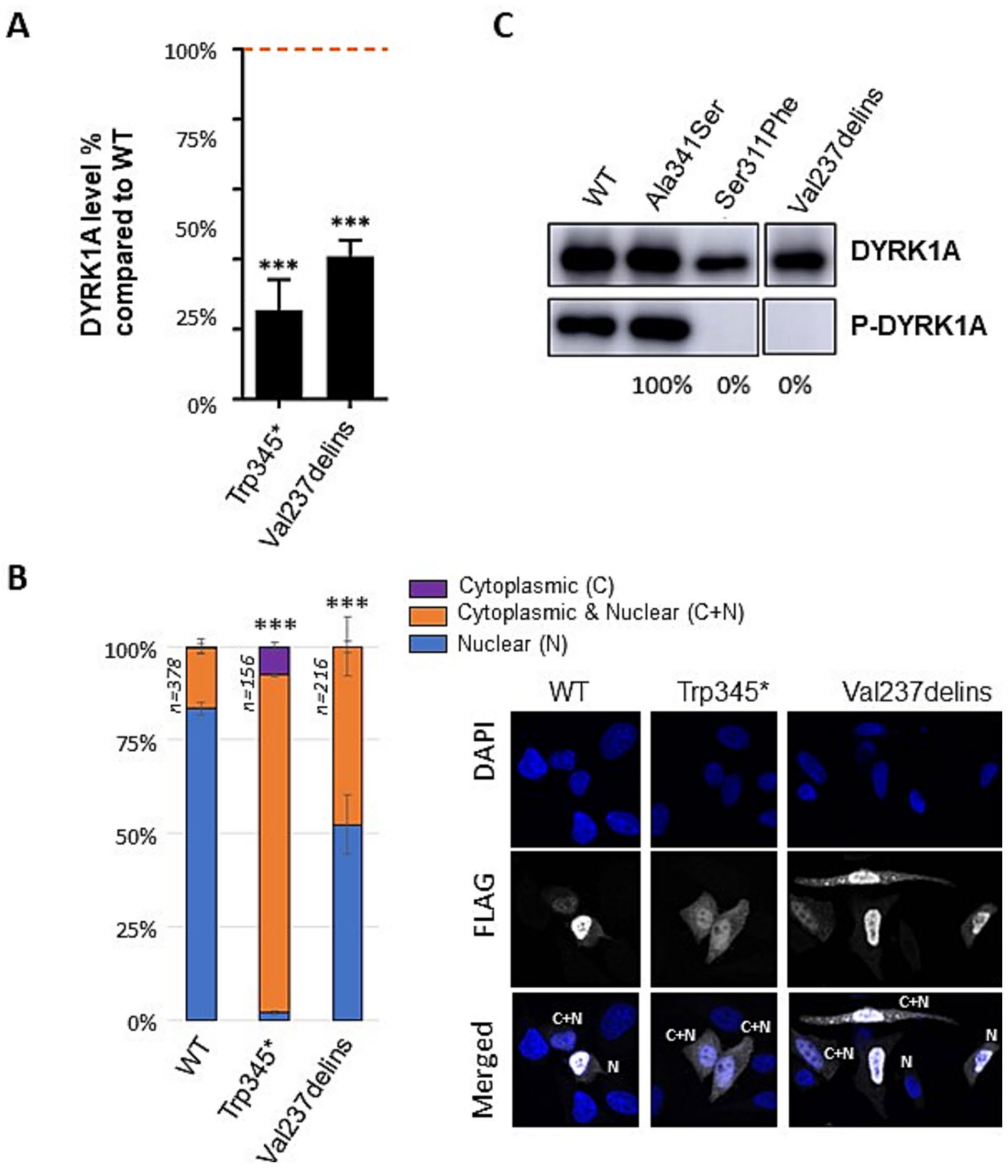
Effect of the p.(Val237_Leu231delinsGlu) (Val237delins); Val237delins) variant on DYRK1A protein level, cellular localization and autophosphorylation. **(A)** Level of mutant DYRK1A proteins expressed in HeLa, HEK293 and COS cells transiently transfected with DYRK1A constructs containing wild-type (WT), Trp345Ter or Val237delins cDNA sequences. Protein levels were normalized on the level of GFP protein (expressed from a cotransfected pEGFP plasmid). Quantifications were performed on a total of *n* = 9 series of cells (*n* = 3 Hela cells, *n* = 3 HEK293 and *n* = 3 COS-7 cells) using ImageJ software. One-way ANOVA with multiple comparison test was performed to compare the level of mutant DYRK1A proteins to the level of WT DYRK1A protein, applying Bonferroni’s correction: ns: not significant; ***p < 0.001; error bars represent SEM, standard error of the mean. **(B)** Cellular localization of DYRK1A WT and mutant proteins observed in HeLa cells after overexpression of FLAG-tagged wild-type and mutant DYRK1A constructs (*n* = 3 series of Hela cells; 50 cells minimum counted per series; the total number of cells counted per condition is indicated in the graph; scale bars of illustrative images correspond to 10 μm). Three types of cellular localization of DYRK1A protein have been observed: DYRK1A located mostly in the nucleus (blue on the graph), in both nucleus and cytoplasm (orange), or mostly in the cytoplasm (purple). Scale bars: 50 μm. Chi-square test was performed to compare localization of mutants and wild-type DYRK1A proteins, ns: not significant; ***p < 0.001; error bars represent SEM, standard error of the mean. Immunofluorescence images illustrate the data (DAPI-labeled nucleus, flag-labeled DYRK1A and merge). **(C)** Measure of DYRK1A autophosphorylation on its Tyr321 tested in HEK293 cells (*n* = 3) transfected with constructs containing WT and Val237delins cDNA sequences, as well two other variants, Ala341Ser which is a benign variant from gnomAD not supposed to affect DYRK1A kinase activity and Ser311Phe which is a kinase-dead variant. An immunoprecipitation with anti-DYRK1A antibody was followed by an immunoblot using anti-phospho-HIPK2 as described in Widowati et al. and Courraud et al. DYRK1A phospho-Tyr321 levels were normalized on DYRK1A total level. The percentage of phosphorylated form for the different mutants compared to the WT proteins is indicated under the blot. One-way ANOVA test was performed to compare mutants to wild-type DYRK1A proteins. ns, not significant; ***p < 0.001; error bars represent SEM, standard error of the mean.

To confirm the loss-of-function consequences of the p.(Val237_Leu241delinsGlu) (also called Val237delins) in-frame variant, we investigated the consequences of this variant *in vitro*. We overexpressed wild-type (WT) and variant DYRK1A proteins in three different cell lines (HEK293, HeLa, COS1) including a known pathogenic missense variant (p.Ser311Pro) as a positive control as well as a benign missense variant (p.Ala341Ser) as negative control. A significant reduction in DYRK1A protein level was observed for the Val237delins variant (Figure 1A). While the wild-type DYRK1A protein is predominantly localized in the nucleus, the mutant variant Val237delins exhibited a significant relocalization to the cytoplasm in a substantial proportion of cells. (Figure 1B). DYRK1A activation requires autophosphorylation at Tyrosine 321 (Himpel et al., 2001). To assess the level of active DYRK1A protein, we detected phospho-DYRK1A (Tyr321) via immunoprecipitation followed by immunoblot using anti-phospho-HIPK2. We demonstrated that the Val237delins variant prevents autophosphorylation (Figure 1C). The effects of the second in-frame deletion/insertion variant of the cohort, p.Ile305_Asp307delinsAsn, was not investigated *in vitro* even when it was first reported (Méjécase et al., 2021). However, the deleterious consequences of a missense variant involving the same amino-acid, Ile305Arg, have been demonstrated previously associating a reduced protein level and an absence of autophosphorylation activity (Courraud et al., 2021).

### 3.4 No obvious NER anomalies observed in fibroblasts of patients with NER-like disorders carrying a pathogenic variant in *DYRK1A*

**Figure 2:**
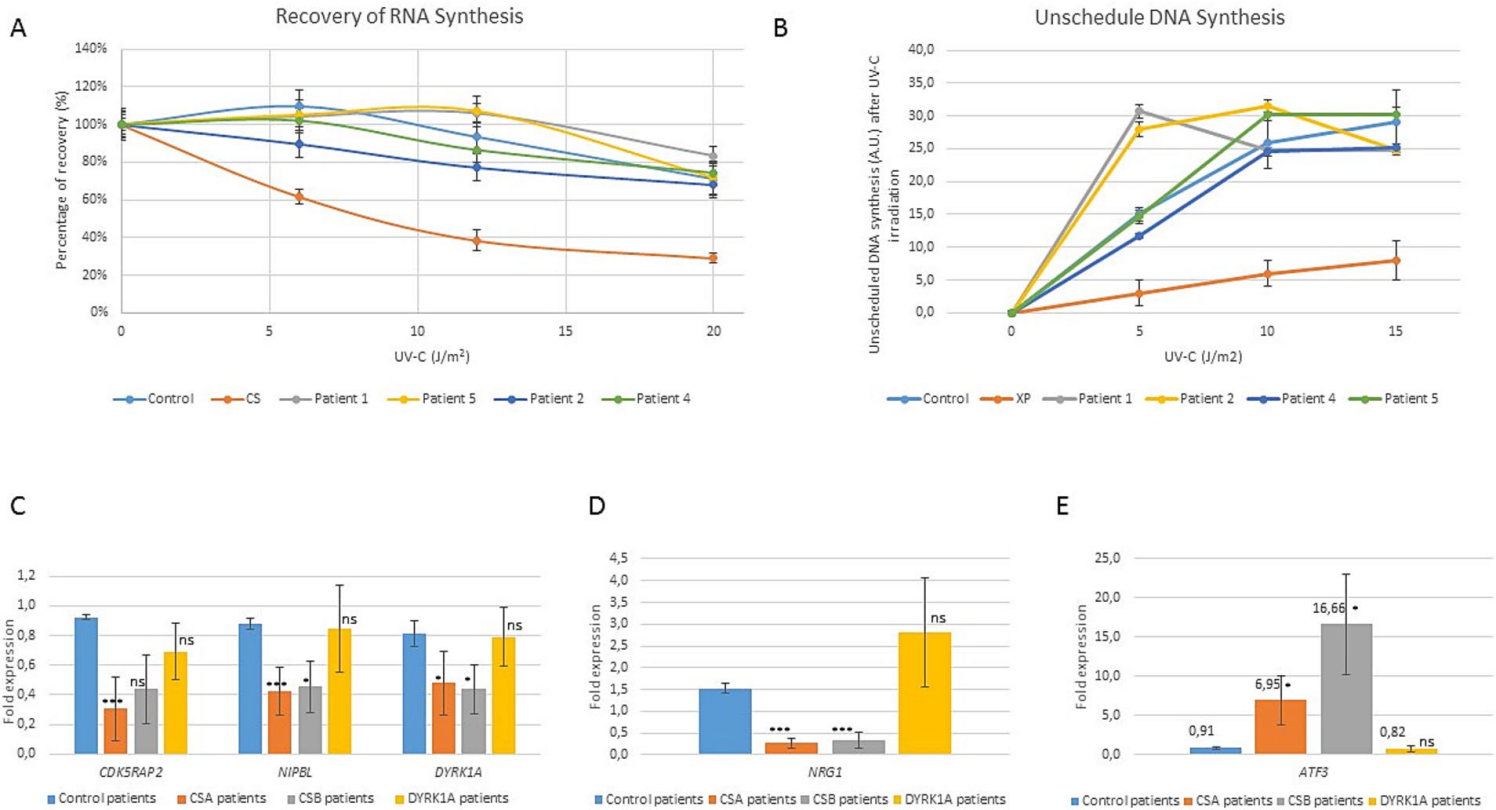
Molecular characterization of DYRK1A patients cell lines compared to Cockayne syndrome patients cell line. **(A)** Recovery of RNA synthesis (RRS) measured 23 h after UV irradiation in fibroblasts from patients 1, 2, 4 and 5, compared to control cell line (individual non affected with CS) and CSA-mutated cell line (CS). Error bars represent SEM, standard error of the mean. **(B)** Unscheduled DNA synthesis (UDS) measured 23 h after UV irradiation in fibroblasts from patients 1, 2, 4 and 5, compared to control cell line (individual non affected with CS) and XPF-mutated cell line (XP). A.U.: arbitrary units. **(C–E)** Study of CS transcriptomic signature and ATF3 expression in *DYRK1A* patients 1, 2, 4 and 5 compared to controls, *CSA* and CSB patients. Expression level of *CDK5RAP2*, *NIPBL, DYRK1A, NRG1*and *ATF3* genes was studied by quantitative RT-PCR in the different cell lines 24 h hours after treatment with 20 J/m^2^ UV-C. Gene expression at 24 h was normalized to 0 h and to *GAPDH* expression. Error bars represent standard deviation. T-test was performed to compare the expression level of each gene in CSA, CSB and *DYRK1A* patients to the expression level in control cell lines, applying Welch’s correction: ns: not significant; *p < 0.005;***p < 0.001.

DYRK1A is known to play a major role in DNA repair, especially homologous recombination (Guard et al., 2019; Menon et al., 2019; Roewenstrunk et al., 2019). To explore the mechanisms underlying the clinical overlap between NER and *DYRK1A* syndrome patients, we evaluated DNA repair assays as well as the CS expression signature on four fibroblastic cell lines from patients with *DYRK1A* pathogenic variants. The Recovery of RNA Synthesis (RRS) test is an assay measuring the ability of cells to restore normal levels of transcription, following ultraviolet (UV) irradiation. This recovery accompanies the elimination of DNA lesions on the transcribed DNA strand by the NER sub-pathway transcription-coupled repair (TCR) and is deficient in CS cells. The unscheduled DNA Synthesis (UDS) assay evaluates the global genome repair ability of the cells after UVC irradiation. All available cell lines of patients with *DYRK1A* variants (patients 1, 2, 4 and 5) exhibited normal DNA repair assays after UV irradiation (Figures 2A,B).

Patients with Cockayne syndrome exhibit a distinct transcriptional signature 24 h post-UV irradiation marked by widespread changes in gene expression, including notable upregulation of transcriptional repressor *ATF3* and downregulation of ATF3-dependent genes, *DYRK1A* being one of the most downregulated ATF3-targeted genes (Epanchintsev et al., 2020). We therefore analyzed by RT-qPCR the expression of *CSB*, *ATF3* and four ATF3-targeted genes (*CDK5RAP2*, *NRG1*, *NIPBL* and *DYRK1A,* which have been described as a signature expression profile for CS) in two control cell lines, 6 CS cell lines (3 CSA linked to *ERCC8* variants and 3 CSB linked to *ERCC6* variants) and the four available *DYRK1A* cell lines. Whereas we confirmed the expected expression signature in CS patients, there was no upregulation of ATF3 nor downregulation of ATF3-dependent genes in *DYRK1A* syndrome cell lines (Figures 2C,D).

## 4 Discussion

Here we report on a cohort of 11 *DYRK1A* patients initially suspected to be affected by nucleotide excision repair disorders. This illustrates the clinical overlap between these disorders that may be connected by disturbance of common molecular pathways.

The *DYRK1A* gene, located on chromosome 21, codes for a serine/threonine dual specificity kinase. Due to the multiplicity of functions performed by its target proteins, DYRK1A is involved in many cellular functions such as cell cycle regulation, apoptosis, synaptic functions, cytoskeletal maintenance and metabolism. DYRK1A is also thought to play a role in the regulation of splicing and transcription. This dosage sensitive protein plays a critical role in neuronal differentiation and brain development (Ananthapadmanabhan et al., 2023).

Haploinsufficiency of the *DYRK1A* gene causes a rare autosomal-dominant intellectual developmental disorder (MRD-7 or DYRK1A syndrome, OMIM #614104). This syndrome manifests with characteristic features such as microcephaly, facial dysmorphism, severe intellectual disability, autism spectrum disorder (ASD), speech impairment and seizures (Bronicki et al., 2015; Ji et al., 2015; van Bon et al., 2016; Evers et al., 2017; Courraud et al., 2021). This condition is now recognized as one of the most frequent genetic causes of intellectual disability (Deciphering Developmental Disorders Study, 2017).

Among the 11 *DYRK1A* variants described in this cohort, four have never been described before. These are 3 truncating variants (p.(Trp345Ter), p.(Val204GlyfsTer7), p.(Val237_Leu241delinsGlu) and p.(Cys235LeufsTer5)) and one in-frame variant p.Val237_Leu241delinsGlu whose pathogenicity has been confirmed by reduced DYRK1A protein expression, reduced nuclear localization and autophosphorylation inability.

Of the 11 variants identified, *de novo* occurrence was confirmed in six cases. The remaining variants, for which segregation analysis was not possible, include four previously reported pathogenic variants and one novel variant (p.(Val237_Leu241delinsGlu)), whose pathogenicity has been validated through functional studies.

Although initially suspected of NER disease, the 11 *DYRK1A* patients of the present cohort are indistinguishable from previously reported *DYRK1A* cases by comparing the clinical DYRK1A score measured on a 20-point scale. Two of the main symptoms observed in *DYRK1A* patients in general and in this cohort in particular, intellectual disability and microcephaly, are also systematically observed in CS patients. Other frequently observed symptoms in this cohort such as feeding troubles and ataxic gait/hypertonia are also commonly found in CS (Supplementary Table S2). The main facial feature shared by the two syndromes is deep-set eyes (Supplementary Figure S1). Despite this striking clinical overlap, some symptoms distinguish the two conditions. *DYRK1A* patients can be distinguished to CS type I patients by a specifically severely impaired language, more frequent seizures and autistic behavior or anxiety. Whereas growth delay is a common feature in both disorders, it is more severe in CS patients (median height −6.62 ± 3.09 SD in a large CS patients cohort (Wilson et al., 2016) compared to −2.6 ± 1.4 DS in the present *DYRK1A* cohort). Moreover, bilateral cataracts and pigmentary retinopathy are more frequent in CS patients. Hearing loss is described for 2 patients in our cohort of 11 patients, while to our knowledge, sensorineural hearing loss had only been reported in one DYRK1A patient previously in the literature (Meissner et al., 2020) and is a common feature in CS. Hair anomalies can be observed in *DYRK1A* syndrome and in TTD (Stefanini et al., 2010; Courraud et al., 2021). Cutaneous photosensitivity is specific to NER disorders (Wilson et al., 2016). Cerebellar and cerebral atrophy together with white matter anomalies and myelination anomalies may be seen in both *DYRK1A* and NER disorders whereas cerebral calcifications typical of CS have not been observed in *DYRK1A* patients (Koob et al., 2016; Courraud et al., 2021).

Other patients initially suspected of having Cockayne syndrome have been reported in the literature. One such case involved a patient with growth delay, psychomotor impairment, photosensitivity, and brain calcifications, who was ultimately diagnosed with Aicardi-Goutières syndrome due to mutations in the *SAMHD1* gene (Senju et al., 2022). More recently, four patients with a Cockayne syndrome-like phenotype, but with a normal response in recovery of RNA synthesis after UV irradiation, were diagnosed with *MORC2*-related disorders (Stafki et al., 2023). Our report highlights that DYRK1A syndrome should now be considered a new differential diagnosis for Cockayne syndrome and included in diagnostic panels for repair disorders.

Transcriptomic signature studied in both CSB and CSA Cockayne patients upon genotoxic (UV) stress has revealed an upregulation of transcriptional repressor *ATF3* and downregulation of ATF3-dependent genes (Kristensen et al., 2013; Epanchintsev et al., 2017, 2020). Interestingly, *DYRK1A* was reported as one of the most down-regulated ATF3-dependent genes in CS fibroblasts. Moreover, several DYRK1A interactome studies have resulted in the discovery of a novel role of DYRK1A in DNA repair (Guard et al., 2019; Menon et al., 2019; Roewenstrunk et al., 2019). We therefore wanted to test whether the cells from our DYRK1A patients had CS cell characteristics. DYRK1A fibroblasts, available for patients 1, 2, 4 and 5, did not exhibit any NER defect (normal RRS and UDS assay), nor *ATF3* upregulation or ATF3-gene downregulation after UV exposure (Figure 2). These results are in accordance with normal UDS assay recently found by Bélanger *et al* in post-UV HeLa cells treated with siRNA against Dyrk1A (Bélanger et al., 2023). Nevertheless the authors do not exclude a non-canonical role of *DYRK1A* in S-phase global-genome repair (Bélanger et al., 2023). An indirect link between DYRK1A and DNA repair has also been demonstrated via phosphorylation regulation of the DREAM complex, a repressor of the various DNA repair systems in *C. elegans* somatic cells. (Bujarrabal-Dueso et al., 2023). Finally, the identification of NER-independent pathways for the resolution of DNA damage on actively transcribed strands suggests new pathophysiological mechanisms to explore, in which the pleiotropic DYRK1A protein could play a role (Bujarrabal-Dueso et al., 2023). Altogether, these results points the fact that DYRK1A is not involved in canonical transcription-coupled repair, the NER sub-pathway impaired in CS. Even if a CS deficit leads to downregulation of DYRK1A, the reciprocal is not the case. DYRK1A is probably involved downstream of CS in the molecular cascade and previously published interactome studies revealed no protein interactions between DYRK1A and CSA nor CSB (Guard et al., 2019; Roewenstrunk et al., 2019). Nevertheless, these common molecular pathways can perhaps explain some similar clinical symptoms that we previously discussed between the two diseases.

To conclude, we reported 11 *DYRK1A* patients initially suspected to be affected by nucleotide excision repair disorders. These two disorders share many symptoms, leading to identified *DYRK1A* haploinsufficiency syndrome as a main differential diagnosis of Cockayne syndrome. We showed that these *DYRK1A* patients were clinically indistinguishable from other *DYRK1A* patients, that *DYRK1A* patients’ cell lines did not exhibit any NER defect and did not share the CS transcriptomic signature. Nevertheless, *DYRK1A* having been identified as a down-regulated gene in CS cells, it could be hypothesized that the clinical overlap relies on the dysregulation of a common molecular pathway, DYRK1A acting downstream of CSA/CSB.

## Data Availability

The datasets presented in this study can be found in online repositories. The names of the repository/repositories and accession number(s) can be found in the article.

## Supplementary material

SUPPLEMENTARY FIGURE S1 Comparison of facial dysmorphia between *DYRK1A* and Cockayne syndromes. **(A)** Patient 4 with *DYRK1A* syndrome. Image previously published in Bronicki et al., 2015 (patient #10). **(B)** CS patient with *ERCC6* mutations at a similar age. DYRK1A syndrome is characterized by bitemporal narrowing, deep-set eyes, a thin upper vermilion and dysplastic ears. In Cockayne syndrome, facial dysmorphia changes over time and is mainly marked by microcephaly and loss of subcutaneous fat, resulting in a protruding nose and sunken eyes.

SUPPLEMENTARY TABLE S1 Primer sequences for ATF3 and ATF3-dependent genes expression level studies.

SUPPLEMENTARY TABLE S2 Detailed clinical features of the *DYRK1A* patients included in the present cohort (Y = presence or N = absence of each sign at latest known visit; NA and empty cells = not available data; SD: standard deviation).

SUPPLEMENTARY TABLE S3 Calculation of the *DYRK1A* clinical score.
