# Supplementary figures and images for: Clinical and molecular overlap between nucleotide excision repair (NER) disorders and DYRK1A haploinsufficiency syndrome
Source: Front Neurosci. 2025 Mar 26;19:1554093. doi: 10.3389/fnins.2025.1554093 (PMC11979163; doi:10.3389/fnins.2025.1554093)

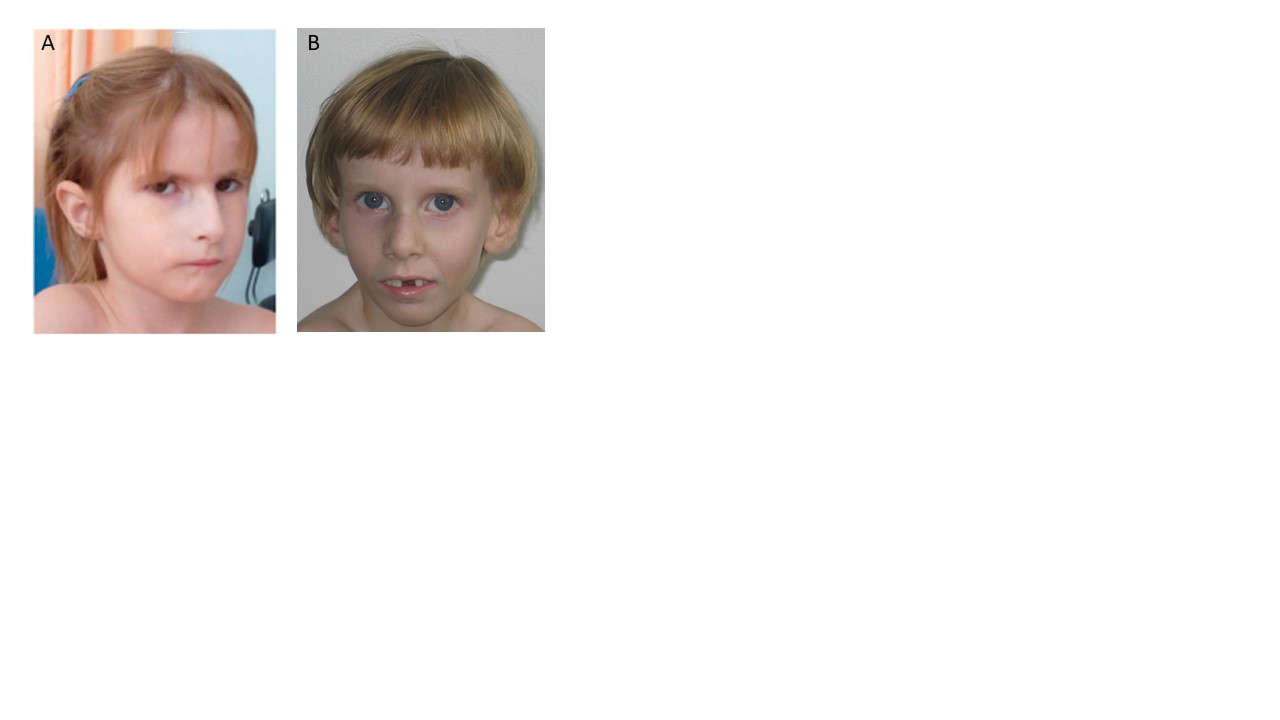

Supplement: SUPPLEMENTARY FIGURE S1 — Comparison of facial dysmorphia between DYRK1A and Cockayne syndromes. (A) Patient 4 with DYRK1A syndrome. Image previously published in Bronicki et al., 2015 (patient #10). (B) CS patient with ERCC6 mutations at a similar age. DYRK1A syndrome is characterized by bitemporal narrowing, deep-set eyes, a thin upper vermilion and dysplastic ears. In Cockayne syndrome, facial dysmorphia changes over time and is mainly marked by microcephaly and loss of subcutaneous fat, resulting in a protruding nose and sunken eyes. [file Image_1.jpeg]
